# Severe COVID-19 Pneumonia in Elderly Patients: Success Rate of Compassioned Use of High Flow Nasal Cannula Therapy

**DOI:** 10.14336/AD.2022.0619

**Published:** 2023-02-01

**Authors:** Isabelle Fabre, Laurène Tardieu, Rachida Ouissa, Ludwig Mounsamy, Bassel Chahim, Pierre-Marie Roger

**Affiliations:** ^1^Infectiologie, Centre Hospitalier Universitaire de Guadeloupe, Guadeloupe, France.; ^2^Faculté de Médecine, Université des Antilles, Guadeloupe, France.; ^3^Gérontologie, Centre Hospitalier Universitaire de Guadeloupe, Guadeloupe, France.; ^4^Service Post-urgence, Centre Hospitalier Universitaire de Guadeloupe, Guadeloupe, France

## Dear Editor,

We read with interest the article by Issa and Soderberg showing that high flow nasal cannula oxygen therapy (HFNO) was feasible in elderly patients with COVID-19 [1]. The outcome of severe COVID-19 pneumonia is particularly bad in elderly patients, with a rate of unfavorable outcome over 50% [2]. This high frequency of patients with respiratory failure due to COVID-19 has led to propose the use of HFNO in medical unit, outside overcrowded intensive care unit (ICU) [3,4]. However, few studies reported its efficiency in elderly patients, and none analyzed the impact of consensual treatments of COVID-19 and related complications [5-8]. Therefore, our aim was to determine the rate of real-life HFNO success for elderly patients admitted for hospital care at a time of major waves of COVID-19 with overcrowded ICUs.

This was a retrospective cohort study conducted in the University Hospital of Guadeloupe (Pointe-à-Pitre, French West Indies). All elderly patients (≥ 65 years old) hospitalized in dedicated wards for a severe COVID-19 pneumonia and requiring HFNO because of a failure of oxygen therapy using a facial mask, were included. Dedicated units for HFNO were put in place mid-2021 in our institution to face successive COVID-19 waves, to have trained staff and to solve technical constraints. To measure the rate of success of HFNO in elderly patients in medical wards, those initially hospitalized in ICU were excluded. RNA detection of the SARS-CoV-2 was assessed by RT-PCR on nasopharyngeal swab. All positive samples were screened for successive variant in a reference lab (Institut Pasteur, Paris, France). Comorbidities were defined by their specific treatments prescribed to the patient before hospital care. Loss of autonomy, including cognitive impairment, dementia whatever the cause, stroke and other neurologic and/or severe psychiatric diseases, was especially reported as it could justify palliative care. The Charlson’s comorbidity index was also used to quantify comorbid conditions. The protocol was approved by the local ethical committee and patients included in the database “French COVID” (recorded center 050). Decisions about HFNO without ICU admission were made in accordance with the patient and/or his relatives on a daily basis with a multidisciplinary team, including physicians from the dedicated wards, plus critical care and palliative care specialists. We reported the entire treatment of severe COVID-19 pneumonia, including steroid, anti-IL-6 therapy, the related preventive or curative treatment of pulmonary embolism, and other complications observed during Covid-19 [8]. All hospitalized patients were treated as following: i) parenteral dexamethasone (6 mg qd) for at least 3 days followed by oral prednisolone (40 mg qd) to achieve a total of 10 days of steroid therapy ii) in the absence of improvement after 24 to 36 hours, tocilizumab was added with one single infusion of 8 mg/kg (maximal dose of 800 mg) iii) prevention of thrombo-embolism used enoxaparine in the absence of renal insufficiency (defined by a creatinine clearance < 30 ml/min). In case of severe renal insufficiency, calciparine was used. We searched for any acute organ failure related to SARS-CoV-2 infection, such as cardiac event, acute kidney injury (defined by a doubling of baseline creatinine values) and neurologic disorders. CT scan analysis was performed respecting the guidelines of the French Society of Thoracic Imaging (e-learning COVID-19: Quantification de l’atteinte parenchymateuse. Société Française d’Imagerie Thoracique. Elearning - Covid 19 en radiologie de A à Z - 27/03/20 | SFR e-Bulletin, accessed 2020 Apr 16). High flow nasal cannula (Airvo2, Fisher-Paykel Healthcare) was usually started with an oxygen flow rate of 40 l/min and FiO2 of 60%, humidified and heated to 31°C. Success of HFNO was defined by respiratory recovery without other mean of oxygen therapy, with a clinical follow-up until hospital discharge. Thereafter, the group HFNO success was compared to other patients (group HFNO failure). The data were analyzed with StatView software version 5.0, and statistical significance was established at P = 0.05. The continuous variables were compared with the Student’s *t*-test or the Mann-Whitney test when appropriate. Proportions were compared with the χ^2^ or Fisher’s exact test when appropriate. Logistic regression was used to study determinants of HFNO success, and the results are presented as adjusted odds ratios (AORs) with their 95% confidence intervals (CIs). Variables were selected for the multivariate analysis based on the level of significance of the bivariate association with HFNO success (p < 0.2). Models were built up sequentially, starting with the variable most strongly associated with HFNO success and continuing until no other variable reached significance or altered the odds ratios of variables already in the model.

**Table 1:** Characteristics of the 101 elderly patients with severe COVID-19 pneumonia treated by HFNO, and risk factor of success using univariate analysis and logistic regression.

Characteristics	Total n = 101 (100%)	HFNO success n = 41 (40.5%)	HFNO failure n = 60 (59.5%)	P	AOR
Third wave March-April 2021 (Alpha variant) Fourth wave July-August 2021 (Delta variant) Fifth wave February-March 2022 (Omicron variant) Sex-ratio (M/F) Age (years) Underlying conditions At least one comorbid condition Diabetes Hypertension Obesity Pulmonary disease Cancer / immunodepression Loss of autonomy Charlson’s comorbid condition index High risk patients (Charlson index > 2) Duration of symptoms before admission Respiration rate on admission (/min) Chest CT scan on admission Lung affected ≤ 25% Lung affected 26-50% Lung affected > 50% Pulmonary embolism *C-reactive protein* (mg/L) on admission Treatment Duration of symptoms at HFNC initiation Tocilizumab Administration differed more than 2 days Other complications due to Covid-19 Acute kidney injury Cardiac complications Suspected or proven bacterial infection Neurologic involvement	25 (25) 50 (49) 26 (26) 1.06 77±6 92 (91) 43 (43) 69 (68) 31 (31) 10 (10) 10 (10) 14 (14) 2.60±1.78 47 (47) 8.4±4.8 32±8 89 (88) 18 (21) 42 (49) 26 (30) 14 (16) 140±89 10.0±4.8 55 (55) 22 (39) 35 (35) 8 (8) 16 (15) 10 (12) 1 (1.1)	8 (20) 18 (44) 15 (37) 2.72 75±7 37 (90) 16 (39) 27 (66) 9 (22) 6 (15) 4 (10) 2 (5) 2.19±1.52 16 (39) 8.9±4.6 33±7 38 (93) 7 (19) 23 (62) 7 (19) 8 (21) 138±87 10.6±4.3 28 (68) 11 (39) 13 (32) 3 (6) 7 (17) 4 (11) 0	17 (28) 32 (54) 11 (18) 0.57 78±6 55 (92) 27 (45) 42 (70) 22 (37) 4 (7) 6 (10) 12 (20) 2.88±1.90 31 (52) 8.1±4.9 31±8 51 (85) 11 (22) 19 (39) 19 (39) 6 (12) 142±92 9.5±5.0 27 (45) 11 (39) 22 (37) 5 (9) 9 (14) 6 (12) 1 (1.8)	0.313 0.351 0.039 < 0.001 0.063 0.805 0.550 0.660 0.115 0.188 0.967 0.030 0.093 0.221 0.268 0.372 0.241 0.690 0.031 0.047 0.233 0.993 0.069 0.021 > 0.999 0.607	4.54 [1.85 - 11.11]

All patients were treated by dexamethasone. HFNO success was defined by hospital discharge alive without mechanical ventilation requirement. All suspected or documented bacterial infections were defined by the administration of an antibiotic therapy.

Over the last three waves of COVID-19 in our territory, 1939 patients were admitted in our institution (mean age 61 years, M/F sex-ratio 1.05), among whom 101 patients (5.2%) older than 64 years benefited of HFNO outside ICU. Their main characteristics are described in Table 1. Most of them (97%) were not vaccinated against SARS-CoV-2. Of note, by the end of the study period, the rate of vaccination in the population of Guadeloupe was 35%. They were mainly admitted in the ID department (n = 93). Comorbid conditions were very frequent, including 14 patients presenting with a loss of autonomy. All patients had a high respiration rate (median 32/min, range [20-50]). Chest CT scan (n = 89) showed pulmonary embolism in 14 patients (16%). COVID-19 also led to other organ dysfunctions including cardiac events (n = 16, including 12 congestive heart failures combined with atrial fibrillation and/or pulmonary edema in 8 cases, and 4 bradycardia), suspected bacterial superinfection leading to an antibiotic therapy (n = 10), acute renal failure (n = 8), and neurologic involvement (n = 1, an encephalitis with transient loss of autonomy).

Among these 10 patients receiving antimicrobial agents, there were one septic shock due *E. coli* bacteremia of urinary source, and one healthcare associated infection related to a peripheral catheter occurring on Day-3. Of note, a prescription of antimicrobial compounds was more observed outside the ID department: 3/8 (37%) *vs* 7/93 (8%), p = 0.034. There was no difference between patients treated without or with antibiotics: mean age 77 years old in both group, comorbid conditions and Charlson’score > 2 (47% *vs* 40% respectively), and severity of the COVID-19 pneumonia on CT scan.

All patients received dexamethasone, and 55 patients also received tocilizumab, 22/55 (39%) having this compound after 48 hours of HFNO. Of note, tocilizumab use increased over time, being prescribed in 0%, 70% and 77% of the cases from the third to the fifth wave.

The success of HFNO was observed in 41 cases, with a mean time of 4.3±3.8 days. The mean duration of hospital stay was 9.2±6.5 days. During hospital stay, circumstances made it possible an ICU admission for 6 patients for mechanical ventilation, among whom 5 ultimately died. Thus, the hospital survival rate for these elderly patients was 41.6% (42/101).

Using a logistic regression, we found that the sole factor associated with HFNO success was the gender: male had a higher chance compared to women, with an adjusted odd ratio [95% CI] of 4.54 [1.85 - 11.11] (see Table 1). As the result of the multivariate analysis was surprising, we compared the characteristics of men and women: Table 2 shows the differences (including trends if p < 0.20) in terms of comorbid conditions, CT scan severity and tocilizumab prescription between gender.

**Table 2:** Main differences between men and women which could explain the better outcome in the former.

Characteristics	Men, n = 52 (51.4%)	Women, n = 49 (48.6%)	P
Comorbid conditions At least one comorbid condition Diabetes Hypertension Obesity Lung affected 26-50% Lung affected > 50% Tocilizumab	45 (87) 18 (35) 32 (62) 9 (17) 26 (58) 9 (20) 32 (62)	47 (96) 25 (51) 37 (76) 22 (45) 16 (39) 17 (41) 23 (47)	0.191 0.095 0.131 0.002 0.082 0.030 0.140

Only trends in univariate analysis are shown (p ≤ 0.20).

Our study shows that, in the context of major waves of COVID-19 with overcrowded ICU, more than 40% of the elderly patients with severe pneumonia and a failure of oxygen therapy using a facial mask, will benefit of HFNO use without ICU requirement. Importantly this rate of favourable outcome using HFNO in elderly patients was obtained when other consensual therapies were prescribed. Gender (male) was the main determinant of HFNO success in multivariate analysis. Besides, we observed a difference between clinical departments in terms of antibiotic use in front of these patients with severe viral pneumonia.

Our study has few limitations. First, in these major waves of COVID-19 infections with overcrowded hospitals, likely patients with the most severe comorbidities were not admitted in HFNO dedicated units. Second, significant differences between men and women appeared in terms of comorbid conditions and other risk factors (see Table 2), limiting conclusion on how gender might influence the outcome of the disease.

Few studies have been published on HFNO use in elderly patients, and most of them had a low number of included patients, and no study reported the associated treatments required for severe COVID-19 pneumonia [5-7]. In a study reporting 32 patients (mean age 79 years) treated with HFNO, the overall survival rate was 25% at hospital discharge [5]. Of note 100% of the patients were treated by cefuroxim plus azithromycin. Another study reported 41 patients (mean age 87 years) treated by HFNO, with a survival in-hospital rate of 17%, steroids and antibiotics being administered to respectively 90% and 93% of the patients [6]. Thus, the high rate of in-hospital survival reported in our study argues for the use an immunosuppressive therapy in elderly patients presenting with severe COVID-19 pneumonia requiring HFNO.

In the logistic regression, gender (male) was associated with HFNO success, and Table 2 indicates that the rate of obesity was 2.6 time smaller in males compared to females. The negative impact of obesity on the outcome of COVID-19 has been largely described, and this comorbid condition is associated with many technical difficulties in front of a respiratory failure [9,10].

Our other result is the low rate of antibiotic therapy prescribed in the ID department in elderly patients with respiratory failure due to SARS-CoV-2, compared to other units of our hospitals and to published data (see above). Several studies have reported that bacterial superinfection of the respiratory tract was uncommon in SARS-CoV-2 infection [11-13]. Also, a recent multicentre study including 914 elderly patients (mean age 86 years) demonstrated that early antibiotic therapy was not associated with a lower rate of 30-day in-hospital death compared to delayed antimicrobials or no prescription (34% *versus* 24%) [14]. Thus, considering the high rate of favourable outcome of HFNO in elderly patients associated with a low rate of antibiotic prescriptions (8% in the ID department), we think that antimicrobials should not be prescribed systematically, and their administration should be preceded of microbial investigations.

Our results could have a significant impact on health strategies and hospital policies, which are still heterogeneous, as the diffusion of HFNO in geriatric departments will conduct to major modifications in the process of care of elderly patients. This perspective implies to improve the oxygen distribution in hospital due to high oxygen volumes required when several HFNO are used on the same hospital area. Further studies should determine the optimal hospital organization allowing HFNO for elderly patients in dedicated units, and the best associated therapies, considering immunosuppressive combination and avoiding systematic antibiotic therapy.

## Data Availability

The data used during the current study is available from the corresponding author on reasonable request
